# Principles of Abdominal Wall Closure: A Narrative Review of Evidence-Based Techniques, Contemporary Updates, and Emerging Innovations

**DOI:** 10.7759/cureus.104512

**Published:** 2026-03-01

**Authors:** Umang K Agrawal, Sakshi Jaiswal

**Affiliations:** 1 General Surgery, ESIC Medical College and Hospital, Varanasi, IND

**Keywords:** abdominal wall closure, component separation, incisional hernia, open abdomen, prophylactic mesh, small-bites technique, surgical site infection

## Abstract

Abdominal wall closure is a critical determinant of postoperative outcomes following laparotomy. Despite advances in surgical technique and perioperative care, complications, such as incisional hernia, wound dehiscence, and surgical site infection (SSI), remain prevalent and contribute significantly to long-term morbidity and healthcare expenditure. Over the past three decades, improved understanding of fascial biomechanics, wound healing physiology, and suture mechanics has reshaped closure strategies. Evidence now supports small bites continuous closure with an adequate suture-to-wound length ratio as a biomechanically superior technique. Selective prophylactic mesh reinforcement in high-risk populations has further reduced hernia incidence. Advances in open abdomen management, component separation techniques, minimally invasive repair, and robotic-assisted closure continue to expand reconstructive options. This narrative review synthesizes anatomical foundations, randomized evidence, preventive strategies, emerging technologies, and complication patterns in abdominal wall closure. Contemporary best practice requires integration of anatomical precision, biomechanical principles, patient risk stratification, and judicious adoption of evolving technologies. Future research should prioritize standardized reporting frameworks, long-term comparative outcomes, and cost-effectiveness analyses.

## Introduction and background

Abdominal wall closure represents one of the most fundamental steps in open abdominal surgery, yet paradoxically, it remains one of the most underestimated determinants of long-term postoperative outcomes. While considerable emphasis is placed on the primary surgical pathology, the integrity of fascial closure ultimately dictates whether patients experience durable recovery or develop complications such as incisional hernia, chronic pain, or wound dehiscence.

Incisional hernia develops in approximately 10-20% of patients after midline laparotomy, and in certain high-risk populations, such as those undergoing emergency surgery, abdominal aortic aneurysm repair, or repeat laparotomy, the incidence may approach 30% [1-3]. Beyond cosmetic concerns, incisional hernias may result in pain, bowel obstruction, impaired mobility, and repeated surgical interventions. Wound dehiscence, though less common, remains a feared complication associated with significant morbidity and mortality, particularly in septic or malnourished patients [4].

Historically, closure techniques were largely dictated by individual surgical training and anecdotal experience. Variability existed in suture type, bite size, stitch interval, and reinforcement strategies. Over the past three decades, however, a more scientific understanding of fascial biomechanics and wound healing has reshaped closure philosophy. Modern abdominal wall closure is no longer viewed as a simple approximation of tissue, but rather as a biomechanically strategic reconstruction of load-bearing anatomy [5].

This review provides a comprehensive synthesis of anatomical foundations, suture mechanics, mesh strategies, open abdomen management, minimally invasive techniques, robotic advances, and complication patterns in abdominal wall closure.

## Review

Methods

A structured narrative review was conducted using PubMed/MEDLINE, Embase, and the Cochrane Library. Literature published between January 1995 and March 2025 was evaluated. Search terms included “abdominal wall closure”, “midline laparotomy”, “small-bites technique”, “suture-to-wound length ratio”, “prophylactic mesh”, “open abdomen”, “component separation”, “laparoscopic ventral hernia repair”, and “robotic abdominal closure”.

Eligible studies included randomized controlled trials, prospective and retrospective cohort studies, meta-analyses, and international guideline statements. Case reports were excluded unless they described novel innovations. Due to heterogeneity in incisional hernia definitions and follow-up durations, a pooled quantitative meta-analysis was not performed. Evidence was synthesized thematically.

Anatomical and biomechanical foundations

The abdominal wall comprises multiple layers, including skin, subcutaneous tissue, fascia, musculature, transversalis fascia, and peritoneum. The linea alba functions as the principal load-bearing structure during midline closure, and principles of optimized midline fascial approximation have been described in foundational closure studies [6,7]. Fascial tissue provides the majority of tensile strength, whereas muscle incorporation contributes little mechanical benefit and may impair perfusion if excessively included.

Wound healing progresses through inflammatory, proliferative, and remodeling phases. During early postoperative healing, tensile strength is reduced and depends largely on suture integrity. Excessive suture tension compromises microvascular circulation and collagen synthesis, predisposing to ischemia and fascial failure [8]. Conversely, inadequate tissue approximation allows separation under physiologic intra-abdominal pressure.

Thus, closure must distribute forces evenly while preserving tissue viability, principles that were emphasized in early analyses of midline laparotomy closure technique [6].

The conceptual evolution of abdominal wall closure strategies is summarized in Table 1.

**Table 1:** Evolution of abdominal wall closure philosophy Source: Adapted from Israelsson and Jonsson [6]

Era	Predominant strategy	Conceptual focus
Traditional Era	Large-bites, interrupted sutures	Mechanical reinforcement
Early Evidence Era	Continuous suturing	Even tension distribution
Modern Evidence-Based Era	Small-bites technique	Biomechanical optimization
Contemporary Era	Selective prophylactic mesh	Risk-stratified prevention
Emerging Era	Robotic precision and biomaterials	Functional and regenerative reconstruction

Small-bites technique

Traditional large-bites closure placed sutures 1-2 cm from the fascial edge. While intuitively strong, this approach concentrates stress at fewer fixation points. The small-bites technique places sutures 5-8 mm from the fascial edge at 5 mm intervals with a suture-to-wound length ratio ≥4:1 [9].

The STITCH randomized trial demonstrated significantly reduced incisional hernia rates with small-bites closure compared to large bites closure [9]. Subsequent meta-analyses confirmed this advantage [10,11].

Biomechanically, small bites increase fixation points, distribute tension more uniformly, and reduce ischemic strangulation. Continuous suturing enhances even load distribution and reduces foreign body burden compared with interrupted sutures [11].

A comparison of traditional and small-bites closure techniques is outlined in Table 2.

**Table 2:** Comparison of large- versus small-bites closure Source: Based on data from Deerenberg et al. [9] and Henriksen et al. [10]

Parameter	Large bites	Small bites
Distance from fascial edge	1–2 cm	5–8 mm
Stitch interval	1–2 cm	5 mm
Suture-to-wound length ratio	Often <4:1	≥4:1
Stress distribution	Uneven, focal	Uniform, distributed
Tissue perfusion	Higher ischemia risk	Better preservation
Incisional hernia risk	Higher	Lower

Prophylactic mesh reinforcement

Certain patients remain at elevated risk despite optimized suturing, and standardized hernia classification systems have facilitated identification of high-risk defect patterns [12]. Obesity increases intra-abdominal pressure; aneurysm patients exhibit collagen abnormalities; emergency surgery compromises tissue integrity.

Pooled evidence from meta-analysis supports prophylactic mesh reinforcement in selected high-risk patients, showing a clinically meaningful reduction in incisional hernia formation [3]. Retro-rectus mesh placement is favored due to improved vascularization and lower infection risk [3,10]. Biologic mesh may be used in contaminated fields, though durability and cost-effectiveness remain debated [13].

Indications for selective prophylactic mesh placement are summarized in Table 3.

**Table 3:** Indications for prophylactic mesh reinforcement Source: Adapted from Jairam et al. [3]

Risk factor	Mechanistic rationale
Obesity (BMI >30)	Elevated intra-abdominal pressure
Abdominal aortic aneurysm	Collagen metabolism abnormality
Emergency laparotomy	Tissue edema and contamination
Recurrent laparotomy	Pre-weakened fascial integrity

Open abdomen and delayed closure

Open-abdomen strategies are utilized in trauma, abdominal compartment syndrome, and severe sepsis [14]. Prolonged fascial retraction results in loss of domain and ventral hernia.

Negative-pressure wound therapy (NPWT) improves delayed fascial closure rates by reducing edema and maintaining medial tension [15]. When closure is not possible, component separation techniques allow medial mobilization while preserving neurovascular supply [16].

Key strategies in open-abdomen management are summarized in Table 4.

**Table 4:** Open-abdomen management strategies Source: Based on Coccolini et al. [14] and Barker et al. [15]

Strategy	Mechanism	Clinical benefit
Negative-pressure wound therapy	Edema control and medial tension	Improved delayed closure rate
Early fascial closure	Restoration of integrity	Reduced ventral hernia
Component separation	Medial advancement of musculature	Closure of large defects

Minimally invasive and robotic-assisted closure

The evolution of minimally invasive surgery has significantly influenced abdominal wall reconstruction. Laparoscopic ventral and incisional hernia repair has become increasingly standardized, supported by international guidelines that demonstrate reduced wound morbidity, shorter hospital stay, and faster recovery when compared with open techniques in selected patients [17].

The introduction of robotic surgical platforms has addressed many technical limitations of conventional laparoscopy. Robotic systems provide enhanced three-dimensional visualization, wristed instrumentation with increased degrees of freedom, tremor filtration, and improved ergonomic control. These features allow more precise intracorporeal suturing, meticulous retrorectus dissection, and accurate mesh placement in anatomically confined spaces.

From a biomechanical perspective, robotic closure enables controlled fascial approximation with reduced tissue trauma. Barbed sutures have been explored in fascial closure and may improve tension distribution and operative efficiency [18].

Despite these advantages, long-term comparative data assessing recurrence, chronic pain, and cost-effectiveness remain limited. Most available studies are observational with short follow-up duration, and randomized comparative trials are required to define the role of robotic-assisted closure relative to optimized open small-bites techniques. In addition, financial cost and training requirements may limit widespread adoption in resource-constrained settings.

Thus, while robotic-assisted abdominal wall closure represents a promising advancement in precision surgery, its definitive role relative to optimized open small-bites closure remains to be established through high-quality randomized trials.

Complications of abdominal wall closure

Complications following abdominal wall closure arise from a complex interplay of patient-related factors, technical variables, and biological healing dynamics. Understanding these mechanisms is essential to prevent long-term morbidity.

Incisional Hernia

Incisional hernia remains the most common late complication of laparotomy [1], and standardized definitions and classification systems have improved outcome reporting across studies. Pathophysiologically, hernia formation results from progressive fascial separation due to mechanical overload exceeding tissue strength. Contributing factors include excessive tension, poor collagen synthesis, obesity, smoking, diabetes, and infection. Importantly, collagen type I to type III ratios may be altered in predisposed patients, particularly those undergoing aortic aneurysm repair.

Clinically, incisional hernias may manifest months to years after surgery and can result in pain, bowel obstruction, cosmetic deformity, and the need for reoperation. Prevention strategies include small-bites continuous closure, maintenance of adequate suture-to-wound length ratio, and selective mesh reinforcement in high-risk patients.

Surgical Site Infection (SSI)

SSI significantly increases the risk of fascial breakdown and subsequent hernia formation [5]. Infection impairs collagen deposition and weakens early wound tensile strength. Even superficial infections may compromise fascial integrity.

Preventive measures include antibiotic prophylaxis, meticulous sterile technique, glycemic control, smoking cessation, and minimization of operative time. In contaminated procedures, closure technique selection and consideration of delayed primary closure may be warranted.

Wound Dehiscence

Wound dehiscence typically occurs within the first postoperative week and may present as partial or complete fascial separation. Risk factors include infection, steroid use, malnutrition, chronic cough, increased intra-abdominal pressure, and technical errors in suturing [4].

Dehiscence represents a surgical emergency when complete and may require reoperation. Ensuring tension-free closure and patient optimization are critical preventive strategies.

Chronic Pain

Chronic postoperative pain may arise from nerve entrapment, mesh-related fibrosis, or excessive scar formation. Precise anatomical awareness during closure, avoidance of nerve injury, and appropriate mesh positioning reduce this risk.

Major complications and preventive strategies are summarized in Table 5.

**Table 5:** Complications of abdominal wall closure Source: Adapted from Luijendijk et al. [1] and van Ramshorst et al. [4]

Complication	Pathophysiology	Timeframe	Prevention strategy
Incisional hernia	Fascial overload and collagen failure	Months–years	Small-bites, mesh reinforcement
Surgical site infection	Impaired collagen deposition	Days–weeks	Antibiotics, sterile technique
Wound dehiscence	Early fascial separation	5–10 days	Tension-free closure
Chronic pain	Nerve entrapment or fibrosis	Months	Anatomical precision

Results

Across the contemporary literature, several consistent patterns emerge regarding abdominal wall closure strategies.

First, randomized trials demonstrate that small-bites continuous closure significantly reduces incisional hernia formation compared with traditional large-bites techniques [9-11]. The magnitude of relative risk reduction is clinically meaningful and reproducible across different patient populations.

Second, across comparative studies, prophylactic mesh reinforcement consistently reduces incisional hernia incidence in high-risk cohorts [3]. Infection rates do not appear significantly elevated when mesh is placed in clean surgical fields and positioned in the retrorectus plane [3,10].

Third, in open-abdomen management, NPWT improves delayed fascial closure rates and reduces ventral hernia formation compared with older temporary closure techniques [14,15]. Early closure remains critical for long-term structural integrity.

Fourth, minimally invasive and robotic approaches demonstrate technical feasibility and improved ergonomic control [17,18]. However, long-term recurrence and cost-effectiveness data remain limited, and definitive superiority over optimized open closure has not yet been established.

Collectively, the evidence supports a hierarchical closure strategy: biomechanically optimized small-bites continuous suturing as baseline standard, risk-stratified prophylactic mesh reinforcement in selected patients, and advanced reconstructive techniques reserved for complex or delayed scenarios.

Discussion

Abdominal wall closure has evolved from a traditional technical maneuver into a biomechanically guided reconstruction process. The small-bites technique demonstrates how incremental technical refinements can produce substantial improvements in long-term outcomes by aligning mechanical distribution with biological healing.

Selective mesh reinforcement reflects preventive surgical thinking, moving from reactive repair toward proactive risk mitigation. This strategy parallels broader surgical shifts toward individualized care.

Open-abdomen management highlights the tension between acute survival and long-term structural restoration. Advances in NPWT and reconstructive techniques have improved closure rates but demand multidisciplinary coordination.

Robotic platforms and advanced biomaterials represent promising innovations; however, cost-effectiveness, reproducibility, and long-term durability must be critically evaluated.

Ultimately, abdominal wall closure should be considered a dynamic integration of anatomy, biomechanics, patient optimization, and evolving technology.

Limitations

This review has several limitations. First, as a narrative review, it does not follow PRISMA methodology and may be subject to selection bias. Second, heterogeneity in study design, patient selection, and incisional hernia definitions limits direct comparison across trials.

Follow-up durations vary significantly, and long-term data beyond five years are limited in many randomized trials. Additionally, robotic and advanced biomaterial data derive primarily from observational studies rather than large randomized trials.

Reporting of suture technique, tension measurement, and mesh plane varies considerably across studies, complicating reproducibility. Patient-related confounders such as smoking, malnutrition, steroid use, and connective tissue disorders are inconsistently controlled.

Future research should prioritize standardized closure reporting frameworks, long-term randomized comparisons, and cost-effectiveness analyses.

## Conclusions

Abdominal wall closure has evolved into a biomechanically informed reconstructive process that directly influences long-term surgical outcomes. Contemporary evidence supports small-bites continuous fascial closure with an adequate suture-to-wound length ratio as the standard technique for midline laparotomy. In selected high-risk populations, prophylactic mesh reinforcement provides additional reduction in incisional hernia incidence. Advances in open-abdomen management, component separation, and minimally invasive and robotic approaches continue to refine reconstructive strategies.

Despite these advances, variability in reporting standards and limited long-term comparative data, particularly for emerging technologies, highlight the need for standardized outcome frameworks and prospective multicenter trials. Abdominal wall closure should be approached as a critical reconstructive phase requiring anatomical precision, biomechanical understanding, and individualized patient risk assessment.
